# Association between Variants in Atopy-Related Immunologic Candidate Genes and Pancreatic Cancer Risk

**DOI:** 10.1371/journal.pone.0125273

**Published:** 2015-05-06

**Authors:** Michelle Cotterchio, Elizabeth Lowcock, Zoe Bider-Canfield, Mathieu Lemire, Celia Greenwood, Steven Gallinger, Thomas Hudson

**Affiliations:** 1 Prevention and Cancer Control, Cancer Care Ontario, Toronto, ON M5G 2L7, Canada; 2 Dalla Lana School of Public Health, University of Toronto, Toronto, ON M5T 3M7, Canada; 3 Ontario Institute for Cancer Research, Toronto, ON M5G 0A3, Canada; 4 Lady Davis Institute for Medical Research, Jewish General Hospital, Montreal, QC H3T 1E2, Canada; 5 Lunenfeld-Tanenbaum Research Institute, Mount Sinai Hospital, Toronto, ON M5G 1X5, Canada; 6 Division of General Surgery, Toronto General Hospital, Toronto, ON M5G 2C4, Canada; 7 Department of Molecular Genetics, University of Toronto, Toronto, ON M5S 1A1, Canada; National Health Research Institutes, TAIWAN

## Abstract

**Background:**

Many epidemiology studies report that atopic conditions such as allergies are associated with reduced pancreas cancer risk. The reason for this relationship is not yet understood. This is the first study to comprehensively evaluate the association between variants in atopy-related candidate genes and pancreatic cancer risk.

**Methods:**

A population-based case-control study of pancreas cancer cases diagnosed during 2011-2012 (via Ontario Cancer Registry), and controls recruited using random digit dialing utilized DNA from 179 cases and 566 controls. Following an exhaustive literature review, SNPs in 180 candidate genes were pre-screened using dbGaP pancreas cancer GWAS data; 147 SNPs in 56 allergy-related immunologic genes were retained and genotyped. Logistic regression was used to estimate age-adjusted odd ratio (AOR) for each variant and false discovery rate was used to adjust Wald p-values for multiple testing. Subsequently, a risk allele score was derived based on statistically significant variants.

**Results:**

18 SNPs in 14 candidate genes (*CSF2*, *DENND1B*, *DPP10*, *FLG*, *IL13*, *IL13RA2*, *LRP1B*, *NOD1*, *NPSR1*, *ORMDL3*, *RORA*, *STAT4*, *TLR6*, *TRA*) were significantly associated with pancreas cancer risk. After adjustment for multiple comparisons, two *LRP1B* SNPs remained statistically significant; for example, *LRP1B* rs1449477 (AA vs. CC: AOR=0.37, 95% CI: 0.22-0.62; p (adjusted)=0.04). Furthermore, the risk allele score was associated with a significant reduction in pancreas cancer risk (p=0.0007).

**Conclusions:**

Preliminary findings suggest certain atopy-related variants may be associated with pancreas cancer risk. Further studies are needed to replicate this, and to elucidate the biology behind the growing body of epidemiologic evidence suggesting allergies may reduce pancreatic cancer risk.

## Introduction

Pancreatic cancer is usually fatal, with fewer than 10% of cases surviving five years [1]. While its etiology is not well understood, smoking [2,3], pancreatitis [4,5], obesity [6] and family history of pancreas cancer [7] are long established pancreatic cancer risk factors, together with the more recently identified ABO blood group [8,9]. There is a growing body of evidence, including meta- and pooled-analyses, that suggests atopic diseases such as allergies and hay fever, are associated with reduced pancreatic cancer risk [10–13]. While there is no accepted understanding of how allergies may reduce pancreatic cancer risk, enhanced cancer immune surveillance has been suggested [10]. In light of the association between atopy and pancreas cancer risk [10–13], and the known inherited component to both atopic conditions [14], and pancreatic cancer [7], it is possible these two diseases may share a common genetic link, and evaluation of the association between atopy-related immunologic genetic variants and pancreatic cancer risk is warranted.

We investigated the association between pancreatic cancer risk and a comprehensive selection of variants in atopy-related immunologic candidate genes to help better understand the observed relationship between allergies and pancreas cancer risk, and to determine whether it may be explained by shared genetic factors.

## Materials and Methods

A population-based case-control study was conducted to investigate the association between variants in atopy-related genes and pancreatic cancer risk. Ethics approval was obtained from the local research ethics boards (REB) (Mount Sinai Hospital REB, Toronto, Canada and University of Toronto REB, Toronto, Canada). Written informed consent was obtained for collection of biologic samples, and each participant had the freedom to decline or withdraw from this study at any given point in time.

### Recruitment of Pancreas Cancer Cases and Controls

Pancreatic cancer cases were recent participants in the Ontario Pancreas Cancer Study, which identified cases through the Ontario Cancer Registry. As described previously [13], the Ontario Pancreas Cancer Study recruited residents of Ontario, Canada with a pathology report confirmed adenocarcinoma of the pancreas, or adenocarcinoma metastasis (confirmed as pancreas cancer by treating physician). Cases diagnosed between February 2011 and August 2012 were eligible for the current study, and of the 1095 pancreatic cancer cases identified, 327 (30%) were deceased or ineligible, and 130 (12%) could not be contacted. Of the 638 cases who were mailed a study package (with consent forms, 4 questionnaires, blood collection form, blood kit), 248 (39%) provided a blood or saliva sample (for DNA extraction) in addition to questionnaires, while 375 completed questionnaires only. Cases took a blood kit to a medical laboratory for their blood draw, and in some instances home blood draws were arranged. Cases that preferred to give a saliva sample were mailed an Oragene DNA Self Collection Kit (DNA Genotek, Kanata, ON) including instructions, consent form, and a pre-paid reply envelope. Participants ranged in age from 30–88 years.

Controls residing in Ontario were recruited during 2011 using a modified random digit dialing procedure and frequency matched to the expected case distribution by sex and 5-year age groups (previously described [13]). Of the 1734 controls who agreed to participate and were mailed study packages, 1285 (74%) returned the questionnaire and blood/saliva request form. Subjects who chose to give a saliva sample were mailed an Oragene DNA Self Collection Kit (DNA Genotek, Kanata, ON) (with instructions, consent forms, and a pre-paid reply envelope). Subjects who chose to give a blood sample were mailed a blood kit which they took to a laboratory under contract with the study for their blood draw. While 1285 controls completed questionnaires, only 608 (47%) provided a blood sample at a laboratory or mailed back a saliva sample (for DNA extraction).

DNA extraction was conducted at the Biospecimen Repository at Mount Sinai Hospital (Toronto, Canada). DNA extraction from blood was conducted using the MaXtract procedure (Qiagen, Valencia, CA), and DNA from saliva was extracted using the Oragene kit methods (DNA Genotek, Kanata, ON). All extracted DNA was stored at 4°C.

### Atopy-Related Immunologic Candidate Gene and SNP Selection

Initially, 183 atopy-related immunologic candidate genes were identified through an extensive literature review of the genetics of atopic diseases, including many observational studies assessing the association between genetic variants and allergy/atopy risk, and many genome-wide association studies (GWAS) of atopy-related traits, plus several cancer studies that assessed immunologic variants, and a few papers about the pathophysiology of atopy (listed in S1 Table). In order to narrow down this list of ~180 candidate genes and select single nucleotide polymorphisms (SNPs) for genotyping, the following screening was undertaken. GWAS datasets from the Pancreatic Cancer Cohort and Case-Control Consortium (PanScan) were downloaded from the Database of Genotypes and Phenotypes (dbGaP) via the National Institute of Health website (phs000206.v3.p2) [15–18]. All SNPs on the Illumina HumanHap550v3.0 array (used in PanScan) that tagged any of our ~180 a priori candidate genes were identified [SNPs within genes of interest (+/- 1kb), or SNPs in high linkage disequilibrium (LD) (R^2^>0.8)]. PLINK v.1.07 [19], the whole genome analysis toolset, was used to estimate associations between pancreatic cancer risk and each tagging SNP. SNPs were sorted according to ascending magnitude of p-value. The most significant SNPs (P<0.01) were selected (retained) for genotyping regardless of minor allele homozygote frequency as reported in HapMap CEU population. SNPs with P>0.01 were not selected for genotyping if the minor allele homozygote frequency was <5%. In cases where SNPs were in high LD (R2>0.8), only the most significant SNPs were retained. The initial 183 genes were pared down to 59 genes. In total, 152 SNPs in 59 immunologic candidate genes were selected to be in the final list for genotyping by the current study (listed in S2 Table).

### DNA Genotyping

Genotyping of DNA from the Ontario cases and controls for the selected SNPs (in 59 genes) was conducted by the Clinical Genomics Centre at Mount Sinai Hospital (Toronto, Canada) using the MassARRAY iPLEX Sequenom Platform (Sequenom, San Diego, CA). Sequenom assay design software was used to design the reaction panels for genotyping the selected SNPs. The software was unable to identify unique optimal primer binding sites for 7 of the 152 SNPs selected, and we identified alternative SNPs in high LD (R^2^ >0.85) for 2 of these 7 SNPs. Therefore, 147 SNPs in 56 genes were included in the final reaction panels for genotyping. There were 10 negative controls and 10 replicates included in the reaction plates. The genotyping success rate was 99%. Call rates were <90% for 16 SNPs, therefore statistical data analyses were restricted to 131 SNPs. Of the 852 samples, 12 failed genotyping (e.g., <60% of SNPs were successfully genotyped). In total, genotype data for 238 cases and 602 controls were available for statistical data analyses.

Genotypes were assessed for significant deviation from Hardy-Weinberg equilibrium using the Online Encyclopedia for Genetic Epidemiology studies calculator [20]; SNP rs3126085 in the *FLG* gene (and 4 other non-significant SNPs) were found to deviate from Hardy-Weinberg equilibrium (at p = 0.01 level of significance).

### Statistical Analysis

Due to concerns of confounding by ethnicity, we restricted all analyses to white persons (results for non-whites (n = 74) are not presented due to small sample size). There were 179 white cases (95 female, 84 male) and 566 white controls (278 female, 288 male) with genotyped DNA that comprise the dataset used for this paper. Odds Ratios (ORs), age group adjusted odds ratios (AORs) and 95% confidence intervals (CI) were estimated using logistic regression. Rare homozygous and heterozygous genotypes were combined when minor allele homozygotes had <5% prevalence among controls and ORs were in the same direction. P-values (Wald test) and p-values adjusted for multiple comparisons were both computed; false discovery rate was used to adjust for multiple comparisons [21]. Analyses were restricted to females for the 8 SNPs in 2 genes on the X chromosome. A risk allele score was derived by summing the total number of risk allele counts for each of the 17 statistically significant SNPs for each person (X chromosome SNP was excluded). Each SNP was scored as 0 or 1 (1 was assigned to individuals carrying 1 or 2 copies of the protective allele), and this was summed up across all 17 SNPs to create the risk allele score. All analyses were conducted using SAS version 9.2 (SAS Institute Inc.).

## Results

Table 1 shows the statistically significant association between variants in 14 atopy-related immunologic genes and pancreas cancer risk (where SNPs had at least one genotype in which the 95% CI did not include 1, or the p-value was less than 0.05). These 14 genes were: colony stimulating factor 2 (*CSF2*), DENN/MADD domain 1B (*DENND1B*), dipeptidyl-peptidase 10 (*DPP10*), filaggrin (*FLG*), interleukin 13 (*IL13*), interleukin 13 receptor alpha 2 (*IL13RA2*), low density lipoprotein receptor-related protein 1B (*LRP1B*), nucleotide-binding oligomerization domain 1 (*NOD1*), neuropeptide S receptor 1 (*NPSR1*), ORM1-like 3 (*ORMDL3*), RAR-related orphan receptor A (*RORA*), signal transducer and activator of transcription 4 (*STAT4*), toll-like receptor 6 (*TLR6*), and T cell receptor alpha (*TRA*). All other candidate gene variants were not found to be statistically significantly associated with pancreas cancer risk (the complete list of variants assessed are listed in S2 Table). Eighteen SNPs in 14 genes were significantly associated with pancreas cancer risk: *CSF2* rs17674015 (CA vs. AA: AOR = 0.58, 95% CI: 0.35–0.97), *DENND1B* rs16841842 (CA/AA vs. CC: AOR = 1.43, 95% CI: 1.02–2.02), *DPP10* rs998429 (CC vs. AA: AOR = 1.96, 95% CI: 1.08–3.56), *FLG* rs3126085 (AG/AA vs. GG: AOR = 1.50, 95% CI: 1.03–2.18), *IL13* rs20541 (CT/TT vs. CC: AOR = 1.48, 95% CI: 1.04–2.10), *LRP1B* rs1449477 (AA vs. CC: AOR = 0.37, 95% CI: 0.22–0.62), *LRP1B* rs2029142 (AA vs. GG: AOR = 2.46, 95% CI: 1.49–4.05), *LRP1B* rs1882164 (AA vs. CC: AOR = 0.51, 95% CI: 0.31–0.83), *LRP1B* rs2052910 (AA vs. CC: AOR = 1.90, 95% CI: 1.07–3.36), *LRP1B* rs10496915 (CA/CC vs. AA: AOR = 1.55, 95% CI: 1.08–2.22), *NOD1* rs2907749 (GA vs. AA: AOR = 0.6, 95% CI: 0.44–0.92), *NPSR1* rs1833090 (AA vs. CC: AOR = 2.13, 95% CI: 1.12–4.03), *ORMDL3* rs7216389 (CC vs. TT: AOR = 0.60, 95% CI: 0.38–0.97), *RORA* rs12913421 (AA vs. GG: AOR = 0.42, 95% CI: 0.19–0.92), *STAT4* rs6738544 (CA vs. CC: AOR = 1.75, 95% CI: 1.20–2.55), *TLR6* rs4833095 (CT vs. TT: AOR = 1.56, 95% CI: 1.08–2.25), *TRA* rs7146411 (AG vs. GG: AOR = 0.66, 95% CI: 0.45–0.99), and *IL13RA2* rs638376 on the X chromosome (GG vs. AA: AOR = 2.20, 95% CI = 1.04–4.67). It is noteworthy that the following 11 SNP associations were in the same direction as found in the PanScan GWAS dataset used to initially screen for variants to retain: rs3126085, rs10496915, rs1449477, rs2052910, rs1882164, rs2029142, rs7216389, rs12913421, rs6738544, rs4833095, rs998429.

**Table 1:** Age-adjusted odds ratios for statistically significant associations between variants in atopy-related candidate genes and pancreas cancer risk.

			Cases		Controls					
Gene	SNP		N	(%)	N	(%)	AOR	95% CI	P	P^1^
CSF2	rs17674015	AA	29	(16)	70	(12)	1.00		0.03	0.44
		CA	67	(37)	279	(49)	0.58	(0.35–0.97)		
		CC	83	(46)	215	(38)	0.92	(0.56–1.53)		
DENND1B	rs16841842	CC	89	(51)	334	(59)	1.00		0.04	0.46
		CA/AA	86	(49)	229	(41)	1.43	(1.02–2.02)		
DPP10	rs998429	AA	93	(52)	327	(58)	1.00		0.08	0.51
		CA	65	(37)	200	(36)	1.15	(0.80–1.65)		
		CC	20	(11)	36	(6)	1.96	(1.08–3.56)		
FLG	rs3126085^2^	GG	121	(69)	430	(77)	1.00		0.04	0.46
		AG/AA	54	(31)	131	(23)	1.50	(1.03–2.18)		
IL13	rs20541	CC	101	(59)	380	(67)	1.00		0.03	0.44
		CT/TT	71	(41)	184	(33)	1.48	(1.04–2.10)		
IL13RA2	rs638376	AA	18	(19)	86	(31)	1.00		0.04	0.46
(females)^3^		GA	58	(61)	145	(53)	2.09	(1.14–3.81)		
		GG	19	(20)	45	(16)	2.20	(1.04–4.67)		
LRP1B	rs1449477	CC	52	(29)	125	(22)	1.00		0.000	0.04
		CA	100	(56)	270	(48)	0.89	(0.60–1.32)		
		AA	26	(15)	169	(30)	0.37	(0.22–0.62)		
LRP1B	rs2029142	GG	38	(21)	202	(36)	1.00		0.001	0.05
		GA	97	(54)	265	(47)	1.99	(1.31–3.02)		
		AA	44	(25)	97	(17)	2.46	(1.49–4.05)		
LRP1B	rs1882164	CC	51	(29)	119	(21)	1.00		0.02	0.44
		CA	87	(50)	279	(50)	0.71	(0.47–1.06)		
		AA	37	(21)	165	(29)	0.51	(0.31–0.83)		
LRP1B	rs2052910	CC	70	(40)	288	(52)	1.00		0.02	0.44
		CA	81	(47)	212	(38)	1.60	(1.11–2.31)		
		AA	22	(13)	51	(9)	1.90	(1.07–3.36)		
LRP1B	rs10496915	AA	108	(63)	407	(72)	1.00		0.02	0.44
		CA/CC	64	(37)	157	(28)	1.55	(1.08–2.22)		
NOD1	rs2907749	AA	98	(55)	259	(46)	1.00		0.05	0.46
		GA	62	(35)	250	(44)	0.64	(0.44–0.92)		
		GG	19	(11)	54	(10)	0.90	(0.51–1.60)		
NPSR1	rs1833090	CC	88	(51)	297	(53)	1.00		0.05	0.46
		CA	67	(39)	238	(42)	0.96	(0.67–1.38)		
		AA	18	(10)	29	(5)	2.13	(1.12–4.03)		
ORMDL3	rs7216389	TT	55	(31)	136	(24)	1.00		0.11	0.60
		TC	81	(46)	266	(47)	0.76	(0.51–1.13)		
		CC	39	(22)	163	(29)	0.60	(0.38–0.97)		
RORA	rs12913421	GG	102	(59)	280	(50)	1.00		0.05	0.46
		GA	62	(36)	229	(41)	0.76	(0.53–1.09)		
		AA	8	(5)	51	(9)	0.42	(0.19–0.92)		
STAT4	rs6738544	CC	53	(30)	230	(41)	1.00		0.01	0.44
		CA	102	(57)	251	(45)	1.75	(1.20–2.55)		
		AA	24	(13)	82	(15)	1.24	(0.72–2.15)		
TLR6	rs4833095	TT	89	(52)	355	(63)	1.00		0.03	0.44
		CT	67	(39)	174	(31)	1.56	(1.08–2.25)		
		CC	15	(9)	34	(6)	1.76	(0.91–3.37)		
TRA	rs7146411	GG	58	(33)	147	(26)	1.00		0.11	0.60
		AG	76	(43)	289	(51)	0.66	(0.45–0.99)		
		AA	43	(24)	126	(22)	0.87	(0.55–1.38)		
Risk Allele Score^4^										
	7–13		69	(43)	154	(29)	1.00		0.0007	
	14–15		72	(44)	251	(47)	0.63	(0.43–0.93)		
	16–17		21	(13)	129	(24)	0.36	(0.21–0.62)		

^1^Adjusted for multiple comparisons (using false discovery rate)

^2^SNP rs3126085 not in Hardy-Weinberg equilibrium

^3^ X chromosome gene, females only

^4^Risk Allele Score: sum of risk allele counts for each of the 17 significant SNPs, each SNP was scored as 1 or 0, and this was summed across SNPs for each person (excluded X chromosome)

After adjustment for multiple comparisons using false discovery rate, two SNPs in the *LRP1B* gene (rs1449477 and rs2029142) remained statistically significant with adjusted p-values <0.05. (These two SNPs have an LD of 0.78, and this is just below our 0.8 cutoff for exclusion). Additionally, the risk allele score was associated with a significant reduction in pancreas cancer risk (score 14–15 vs. 7–13 (referent): AOR = 0.63, 95% CI: 0.43–0.93 and score 16–17 vs. 7–13: AOR = 0.36, 95% CI: 0.21–0.62; p = 0.0007).

## Discussion

There is a paucity of literature with which to compare our findings as this is the first study to comprehensively evaluate the association between variants in allergy-related immunologic candidate genes and pancreatic cancer risk. While many studies have examined the association between a history of allergies and pancreas cancer risk, few have evaluated variants in allergy-related genes and pancreatic cancer risk. In 2005, a meta-analysis of ten case-control and four cohort studies reported that allergies, such as hay fever, were associated with a statistically significant reduction in pancreatic cancer risk [10]. In 2014, Cotterchio et al. [13] reported that allergies, such as hay fever, were associated with a significant reduction in pancreatic cancer risk, and this was greatest for those whose skin prick test was positive for allergens. These findings support the growing body of evidence suggesting allergies are associated with reduced pancreatic cancer risk.

One small case-control study evaluated the association between variants in interleukin-4 (*IL-4*) and *IL-4-receptor* genes and pancreatic cancer risk, and no association was observed [22]; although limitations included insufficient power, and no other genes relevant to allergies were studied. A recent pathway-based analysis of pooled GWAS data reported that T-helper (Th) immune response genes (Th1/Th2), such as transforming growth factor-beta receptor 2 (*TGFBR2*), chemokine ligand 18 (*CCL18*), and *IL13RA2*, were associated with pancreatic cancer risk [23].

We found 18 SNPs in 14 atopy-related genes (*CSF2*, *DENND1B*, *DPP10*, *FLG*, *IL13*, *IL13RA2*, *LRP1B*, *NOD1*, *NPSR1*, *ORMDL3*, *RORA*, *STAT4*, *TLR6*, *TRA*) were significantly associated with pancreas cancer risk. After adjustment for multiple comparisons, two SNPs in the *LRP1B* gene remained statistically significant. Furthermore, the derived risk allele score was significantly associated with reduced pancreatic cancer risk.

*LRP1B* (on chromosome 2) belongs to the low density lipoprotein receptor gene family. *LRP1B* binds with multiple ligands and is involved in the clearance of ligands, and also regulates many processes such as lipid metabolism, and nutrient transport [24–27]. *LRP1B* has been implicated in antigen presentation and as a regulator of inflammation and progression in cancer, with the involvement of LRP1-deficient macrophages (as reviewed in reference [28]). Although most of these *LRP1B* functions have been defined in different studies, it is interesting to see that inflammation, a core process of asthma and cancer, is now also genetically associated with both asthma [29] and pancreatic cancer (this study) at the *LRP1B* locus. It is also possible that the biologic mechanism responsible for the association we observed between *LRP1B* and pancreas cancer risk is unrelated to the immune system. For example, it has been suggested that *LRP1B* may be a tumor suppressor gene implicated in gastric cancer [24], and inactivation of *LRP1B* may enhance thyroid cancer growth by modulating the extracellular environment [30]. Additionally, a large study of somatic copy-number alterations and human cancers identified *LRP1B* deletions as a significant finding [31].

In conclusion, our findings suggest an association between several atopy-related genes and pancreatic cancer risk, and this somewhat supports the possibility that the reduced pancreatic cancer risk associated with allergic diseases may be partly explained by shared genetic factors. Understanding the association between atopic diseases and reduced pancreas cancer risk is of importance and further research is needed to elucidate the biology behind the growing body of epidemiologic evidence suggesting allergies may reduce pancreatic cancer risk. It has been suggested that once the relationship between the immune system and pancreas cancer is better understood then stimulation of the immune system, antibody therapy and vaccines may be possible avenues to explore for pancreas cancer prevention. In addition, larger epidemiologic studies are needed to replicate our findings, and to provide the power to definitely rule out the association between pancreatic cancer risk and the hundreds of other immunologic genes. Lastly, biological studies of the implicated genes and their respective proteins and pathways are needed to understand the role of allergic and immune processes in the development of pancreas cancer.

## Supporting Information

S1 Table Immunologic candidate genes identified in the literature as being involved in atopic diseases such as allergies and asthma (n = 183 genes). (DOCX)

S2 Table Atopy-related immunologic candidate genes/SNPs identified in the literature (see S1 Table) and subsequently selected (retained) for genotyping based on preliminary GWAS findings using pancreas cancer dbGaP datasets (n = 59 genes, 152 SNPs). (DOCX)
