# Supplementary material for: Association between Variants in Atopy-Related Immunologic Candidate Genes and Pancreatic Cancer Risk
Source: PLoS One. 2015 May 6;10(5):e0125273. doi: 10.1371/journal.pone.0125273 (PMC4422524; doi:10.1371/journal.pone.0125273)
Supplement: S1 Table — (DOCX) [file pone.0125273.s001.docx]

**Supporting Information Table S1.** Immunologic candidate genes identified in the literature as being involved in atopic diseases such as allergies and asthma (n=183 genes)

| **Genes associated with atopy in observational epidemiologic studies (+/- GWAS):** | |
| --- | --- |
| ADAM33 | (Blumenthal et al. 2006; Ober and Hoffjan 2006; Su et al. 2008) |
| ADRB2 | (Giubergia et al. 2009; Ober and Hoffjan 2006; Woszczek et al. 2005) |
| C3 | (Barnes et al. 2006; Inoue et al. 2008; Purwar et al. 2009) |
| C5 | (Hasegawa et al. 2004; Ricci et al. 2011) |
| CCL11 | (Batra et al. 2007; Shin et al. 2003; Wang et al. 2007) |
| CCL26 | (Chae et al. 2005; Owczarek et al. 2010) |
| CCL5 | (Fryer et al. 2000; Kim et al. 2004; Leung et al. 2005; Ober and Hoffjan 2006) |
| CCR2 | (Nakamura et al. 2007) |
| CCR3 | (Azazi et al. 2007; Nakamura et al. 2007) |
| CD14 | (Han et al. 2010; Litonjua et al. 2005; Micheal et al. 2011; Ober and Hoffjan 2006) |
| CHI3L1 | (Kwon et al. 2011; Sohn et al. 2009) |
| CMA1 | (Hersberger et al. 2010; Sharma et al. 2005; Weidinger et al. 2005A) |
| CSF1R | (Andiappan et al. 2011; Shin et al. 2010) |
| CSF2RB | (Johnson et al. 2011) |
| CTLA4 | (Munthe-Kaas et al. 2004; Ober and Hoffjan 2006; Yang et al. 2004) |
| DPP10 | (Allen et al. 2003; Gao J. et al. 2010; Michel et al. 2010; Zhou et al. 2009) |
| EDN1 | (Immervoll et al. 2001; Michel et al. 2010, Zhu et al. 2008) |
| EPX | (Hrdlickova and Izakovicova-Holla 2009; Nakamura et al. 2003) |
| FCER1A | (Granada et al. 2012; Potaczek et al. 2007; Weidinger et al. 2008; Zhou et al. 2012) |
| FCER2 | (Chan et al. 2011; Tantisira et al. 2007) |
| FCGR2A | (Zeyrek et al. 2008) |
| FLG | (van den Oord and Sheikh 2009; Poninska et al. 2011; Schuttelaar et al. 2009) |
| FOXJ1 | (Li et al. 2006) |
| FOXP3 | (Fodor et al. 2011; Suttner et al. 2010; Zhang, et al. 2009A) |
| GATA3 | (Arshad et al. 2008; Zhang et al. 2009B) |
| GSTM1 | (Gerbase et al. 2011; Gilliland et al. 2004; Ober and Hoffjan 2006) |
| GSTP1 | (Gerbase et al. 2011; Ober and Hoffjan 2006; Reddy et al. 2010) |
| GSTT1 | (Minelli et al. 2010; Ober and Hoffjan 2006) |
| HAVCR1 | (McIntire et al. 2004; Mou et al. 2010) |
| HLA-A | (Granada et al. 2012; Lee et al. 2001) |
| HLA-DQA1 | (Ober and Hoffjan 2006; Wang et al. 2004) |
| HLA-DQA2 | (Andiappan et al. 2011; Granada et al. 2012) |
| HLA-DQB1 | (Andiappan et al. 2011; Kalpaklioğlu and Turan 2002; Munthe-Kaas et al. 2007; Ober and Hoffjan 2006) |
| HLA-DRB1 | (Andiappan et al. 2011; Kalpaklioğlu and Turan 2002; Moffatt et al. 2010; Ober and Hoffjan 2006) |
| HLA-G | (Ciprandi et al. 2009; Granada et al. 2012; Ober and Hoffjan 2006) |
| IFNG | (Barnes et al. 1999; Hussein et al. 2009; Nakao et al. 2001; Ober and Hoffjan 2006) |
| IL10 | (Hussein et al. 2011; Imboden et al. 2006; Muller et al. 2009; Ober and Hoffjan 2006) |
| IL13 | (Bunyavanich et al. 2011A; Granada et al. 2012; Johnson et al. 2011; Ober and Hoffjan 2006) |
| IL18 | (Imboden et al. 2006; Kruse et al. 2003; Nieters et al. 2004) |
| IL1A | (Joki-Erkkilä et al. 2003; Karjalainen et al. 2002) |
| IL1B | (Joki-Erkkilä et al. 2003; Zeyrek et al. 2008) |
| IL1RL1 | (Gudbjartsson et al. 2009; Reijmerink et al. 2010; Shimizu et al. 2005) |
| IL2 | (Christensen et al. 2006; Nieters et al. 2004; Ramasamy et al. 2011) |
| IL21 | (Chatterjee et al. 2009; Hiromura et al. 2007; Kobayashi et al. 2009) |
| IL21R | (Hecker et al. 2003; Pène et al. 2006) |
| IL33 | (Moffatt et al. 2010; Sakashita et al. 2008) |
| IL4 | (Hersberger et al. 2010; Imboden 2006; Li et al. 2008; Lu et al. 2011; Ober and Hoffjan 2006) |
| IL4R | (Genuneit et al. 2009; Johnson et al. 2011; Nieters et al. 2004; Ober and Hoffjan 2006) |
| IL5 | (Gudbjartsson et al. 2009; Kabesch et al. 2007) |
| IL5RA | (Cheong et al. 2005; Namkung et al. 2007) |
| IL6 | (Imboden et al. 2006; Nieters et al. 2004) |
| LTA | (Ober and Hoffjan 2006; Sharma et al. 2006; Trabetti et al. 1999) |
| LTC4S | (Eskandari et al. 2006; Moissidis et al. 2005; Ober and Hoffjan 2006) |
| MS4A2 | (Daniels et al. 1996; Nagata et al. 2001; Ober and Hoffjan 2006; Potaczek et al. 2007) |
| MTHFR | (Husemoen et al. 2006; Thuesen et al. 2010) |
| NOD1 | (Eder et al. 2006; Ober and Hoffjan 2006; Ramasamy et al. 2011; Weidinger et al. 2005 B) |
| NOS1 | (Hollá et al. 2004; Immervoll et al. 2001; Ober and Hoffjan 2006) |
| NOS2 | (Holla et al. 2006; Konno et al. 2001) |
| NOS3 | (Djidjik et al. 2007; Holla et al. 2002) |
| NPSR1 | (Andiappan et al. 2011; Castro-Giner et al. 2010; Ober and Hoffjan 2006) |
| ORMDL3 | (Moffatt et al. 2010; Ober 2011) |
| PHF11 | (Gao J. et al. 2010; Jang et al. 2005; Ober and Hoffjan 2006; Zhang et al. 2003) |
| PTGDR | (Isidoro-García et al. 2011; Sanz et al. 2006) |
| PTGS2 | (Chan et al. 2007; Ober and Hoffjan 2006; Shi et al. 2008) |
| RAD50 | (Li et al. 2010; Murk et al. 2011; Weidinger et al. 2008) |
| RNase3 | (Jönsson et al. 2010; Kang et al. 2010) |
| SCGB1A1 | (Candelaria et al. 2005; Ku et al. 2011; Ober and Hoffjan 2006) |
| SOCS1 | (Mostecki et al. 2011) |
| SPINK5 | (Hubiche et al. 2007; Ober and Hoffjan 2006; Walley et al. 2001) |
| STAT1 | (Hattori et al. 2007; Pinto et al. 2007) |
| STAT6 | (Amoli et al. 2002; Granada et al. 2012; Ober and Hoffjan 2006; Weidinger et al. 2008) |
| TAP1 | (Ismaïl et al. 1997; Kim et al. 2007; Ober and Hoffjan 2006) |
| TBXA2R | (Leung et al. 2002; Ober and Hoffjan 2006; Shin et al. 2003) |
| TGFB1 | (Li et al. 2007; Meng et al. 2005; Ober and Hoffjan 2006) |
| TGFB2 | (Hatsushika et al. 2007; Zhang et al. 2008) |
| TLR2 | (Eder et al. 2004; Kormann et al. 2009; Ober and Hoffjan 2006; Oh et al. 2009; Qian et al. 2010) |
| TLR4 | (Genuneit et al. 2009; Krasznai et al. 2011; Ober and Hoffjan 2006; Senthilselvan et al. 2008) |
| TLR6 | (Miedema et al. 2011; Ober and Hoffjan 2006; Ramasamy et al. 2011) |
| TNF | (Krasznai et al. 2011; Minhas et al. 2010; Ober and Hoffjan 2006; Sharma et al. 2006) |
| TSLP | (Bunyavanich et al. 2011B; Harada et al. 2011; Ramasamy et al. 2011) |
| VDR | (Michel et al. 2010; Poon et al. 2004; Raby et al. 2004) |
| **Genes associated with atopy in GWAS alone:** | |
| ABL2 | (Ramasamy et al. 2011) |
| ADRA1B | (Mathias et al. 2010) |
| ANKRD46 | (Wan et al. 2011) |
| C1orf30 and LRRC32 | (Ramasamy et al. 2011) |
| C16orf72 | (Ramasamy et al. 2011) |
| CA10 | (Li et al. 2010) |
| CLEC16A | (Ramasamy et al. 2011) |
| DENND1B | (Sleiman et al. 2010) |
| DNAH5 | (Ramasamy et al. 2011) |
| DNAJC6 | (Andiappan et al. 2011) |
| ENTPD6 | (Ramasamy et al. 2011) |
| EPS15 | (Ramasamy et al. 2011) |
| FNDC3A | (Wan et al. 2011) |
| GATA2 | (Gudbjartsson et al. 2009) |
| GFRA2 | (Gudbjartsson et al. 2009) |
| GLI3 | (Ramasamy et al. 2011) |
| GNA13 | (Mathias et al. 2010) |
| HLA region | (Ramasamy et al. 2011) |
| IKZF2 | (Gudbjartsson et al. 2009) |
| IL2RB | (Moffatt et al. 2010) |
| KCNJ2 | (Li et al. 2010) |
| KIRREL3 | (Wan et al. 2011) |
| LRP1B | (Li et al. 2010) |
| MRPL4 | (Andiappan et al. 2011) |
| MYB | (Gudbjartsson et al. 2009) |
| Near CROCC | (Ramasamy et al. 2011) |
| Near PPM1A and DHRS7 | (Ramasamy et al. 2011) |
| NETO1 | (Wan et al. 2011) |
| OAT | (Wan et al. 2011) |
| PDE4D | (Himes et al. 2009) |
| PIK3AP1 | (Andiappan et al. 2011) |
| PRNP | (Mathias et al. 2010) |
| PYHIN1 | (Torgerson et al. 2011) |
| RORA | (Moffatt et al. 2010; Torgerson et al. 2011) |
| RTP2 | (Torgerson et al. 2011) |
| SEMA6A | (Ramasamy et al. 2011) |
| SH2B3 | (Gudbjartsson et al. 2009) |
| SLC22A5 | (Moffatt et al. 2010) |
| SLC25A46 | (Ramasamy et al. 2011) |
| SMAD3 | (Moffatt et al. 2010; Torgerson et al. 2011) |
| SNX10 | (Li et al. 2010) |
| STK35 | (Wan et al. 2011) |
| TLE4 | (Hancock et al. 2009) |
| TMEM108 | (Ramasamy et al. 2011) |
| TMEM232 | (Ramasamy et al. 2011) |
| UBXN2B | (Wan et al. 2011) |
| WDR36 | (Gudbjartsson et al. 2009) |
| **Genes encoding proteins that play a role in atopy, though not yet evaluated in human genetic association studies:** | |
| CYSLTR1 | (Canonica 2002) |
| CYSLTR2 | (Canonica 2002) |
| CCL17 | (Barnes 2011) |
| CCL18 | (Chang et al. 2010; Hon et al. 2011) |
| CCL22 | (Barnes 2011) |
| CCL3 | (Di Sciascio et al. 2007) |
| CCL4 | (Benson et al. 2006) |
| CCR4 | (Barnes 2011) |
| CD28 | (Barnes 2011) |
| CD40 | (Corry and Kheradmand 1999; Hattori et al. 2007) |
| CD40LG | (Pawankar et al. 1997) |
| CD80 | (Corry and Kheradmand 1999) |
| CD86 | (Takizawa et al. 2007) |
| CRLF2 | (Yu et al. 2010) |
| CSF2 | (Benson et al. 2009) |
| FCER1G | (Sääf et al. 2008) |
| HAVCR2 | (Bossé and Hudson 2007) |
| HRH1 | (Shirasaki et al. 2012) |
| HRH4 | (Broide 2010) |
| ICAM1 | (Braunstahl et al. 2001) |
| ICOS | (Botturi et al. 2011; Shilling et al. 2005) |
| IL12RB1 | (Barnes 2011) |
| IL12RB2 | (Barnes 2011; Yokoe et al. 2001; Kondo et al. 2001) |
| IL13RA1 | (Rothenberg et al. 2011) |
| IL13RA2 | (Hsi et al. 2011) |
| IL17A | (Wisniewski and Borish 2011) |
| IL17RB | (Wang and Liu 2009) |
| IL22 | (Souwer et al. 2010) |
| IL25 | (Broide 2010; Wang and Liu 2009) |
| IL27 | (Barnes 2011) |
| IL7R | (Gao PS et al. 2010; Mobini et al. 2009) |
| IL9 | (Williams et al. 2012) |
| KIT | (Barnes 2011) |
| KITLG | (Barnes 2011) |
| LTB4R | (Barnes 2011) |
| MAF | (Naito et al. 2011) |
| NFATC1 | (Wisniewski and Borish 2011) |
| NFATC2 | (Wisniewski and Borish 2011) |
| NFATC3 | (Wisniewski and Borish 2011) |
| NFATC4 | (Wisniewski and Borish 2011) |
| NFKB1 | (Corry and Kheradmand 1999) |
| NFKB2 | (Corry and Kheradmand 1999; Martino et al. 2012) |
| P2RX7 | (Barnes 2011) |
| PRG2 | (Pawankar et al. 2011) |
| PTGDR2 | (Barnes 2011; Shirasaki et al. 2009) |
| STAT4 | (Wisniewski and Borish 2011) |
| TBX21 | (Wisniewski and Borish 2011) |
| TGFB3 | (Salib et al. 2004) |
| TGFBR1 | (Salib 2007; Salib et al. 2004) |
| TGFBR2 | (Bottema et al. 2010; Chen et al. 2010; Salib et al. 2004) |
| TNFRSF4 | (Wang and Liu 2009) |
| TNFSF4 | (Wang and Liu 2009) |
| TRA | (Barnes 2011) |
| TRB | (Barnes 2011) |
| VCAM1 | (Braunstahl et al. 2001) |

Note: Literature review conducted during 2012.

**References for S1 Table:**

Allen M, Heinzmann A, Noguchi E, Abecasis G, Broxholme J, Ponting CP, et al. Positional cloning of a novel gene influencing asthma from Chromosome 2q14. Nature Genetics. November 2003; 35(3): 258-263.

Amoli MM, Hand S, Hajeer a H, Jones KP, Rolf S, Sting C, et al. Polymorphism in the STAT6 gene encodes risk for nut allergy. Genes and Immunity. 2002; 3: 220–224.

Andiappan AK, Wang DY, Anantharaman R, Parate PN, Suri BK, Low HQ, et al. Genome-Wide Association Study for Atopy and Allergic Rhinitis in a Singapore Chinese Population. PLoS ONE. 2011; 6(5): e19719. doi:10.1371/journal.pone.0019719

Arshad SH, Karmaus W, Kurukulaaratchy R, Sadeghnejad A, Huebner M, Ewart S.Polymorphisms in the interleukin 13 and GATA binding protein 3 genes and the development of eczema during childhood. Br J Dermatol. 2008; 158(6):1315–1322.

Azazi EA, Bakir SM, Mohtady HA, Almonem AA. Circulating chemokine eotaxin and chemokine receptor CCR3 in allergic patients. Egypt J Immunol. 2007; 14(2):73-82.

Barnes KC, Freidhoff LR, Nickel R, Chiu YF, Juo SH, Hizawa N, et al. Dense mapping of chromosome 12q13.12-q23.3 and linkage to asthma and atopy. Allergy Clin Immunol. 1999;104:485-91.

Barnes KC, Grant a V, Baltadzhieva D, Zhang S, Berg T, Shao L, et al. Variants in the gene encoding C3 are associated with asthma and related phenotypes among African Caribbean families. Genes and Immunity. 2006; 7: 27-35.

Barnes PJ. Pathophysiology of allergic inflammation. Immunological Reviews. 2011; 242: 31-50.

Batra J, Rajpoot R, Ahluwalia J, Devarapu SK, Sharma SK, Dinda AK, et al. A hexanucleotide repeat upstream of eotaxin gene promoter is associated with asthma, serum total IgE and plasma eotaxin levels. J Med Genet. 2007; 44:397-403.

Benson M, Mobini R, Barrenäs F, Halldén C, Naluai AT, Säll T, et al. A haplotype in the inducible T-cell tyrosine kinase is a risk factor for seasonal allergic rhinitis. Allergy. 2009; 64(9):1286-91.

Benson M, Langston MA, Adner M, Andersson B, Torinssson-Naluai Å, Cardell LO. A network-based analysis of the late-phase reaction of the skin. J Allergy Clin Immunol. 2006; 118:220-5.

Bottema RWB, Kerkhof M, Reijmerink NE, Thijs C, Smit HA, van Schayck CP, et al. Gene-gene interaction in regulatory T-cell function in atopy and asthma development in childhood. J Allergy Clin Immunol. 2010;126:338–46, 346.e1–10.

Blumenthal MN, Langefeld CD, Barnes KC, Ober C, Meyers DA, King RA, et al. A genome-wide search for quantitative trait loci contributing to variation in seasonal pollen reactivity. J Allergy Clin Immunol. 2006; 117:79-85.

Botturi K, Lacoeuille Y, Cavaillès A, Vervloet D, Magnan A. Differences in allergen-induced T cell activation between allergic asthma and rhinitis: Role of CD28, ICOS and CTLA-4. Respir Res. 2011;12:25.

Bossé Y, Hudson TJ. Toward a comprehensive set of asthma susceptibility genes. Annu Rev Med. 2007; 58:171–84.

Braunstahl GJ, Overbeek SE, Kleinjan A, Prins JB, Hoogsteden HC, Fokkens WJ. Nasal allergen provocation induces adhesion molecule expression and tissue eosinophilia in upper and lower airways. J Allergy Clin Immunol. 2001;107(3):469-76.

Broide DH. Allergic rhinitis: Pathophysiology. Allergy Asthma Proc. 2010; 31:370-374.

Bunyavanich S, Shargorodsky J, Celedón JC. A meta-analysis of Th2 pathway genetic variants and risk for allergic rhinitis. Pediatric Allergy Immunology. 2011A; 22:378–387.

Bunyavanich S, Melen E, Wilk JB, Granada M, Soto-Quiros ME, Avila L, et al. Thymic stromal lymphopoietin (TSLP) is associated with allergic rhinitis in children with asthma. Clinical and Molecular Allergy. 2011B; 9:1.

Candelaria PV, Backer V, Laing I a, Porsbjerg C, Nepper-Christensen S, de Klerk N, et al. Association between asthma-related phenotypes and the CC16 A38G polymorphism in an unselected population of young adult Danes. Immunogenetics. 2005; 57:25-32.

Canonica GW. Introduction to nasal and pulmonary allergy cascade. Allergy. 2002; 57: Suppl. 75: 8-12.

Castro-Giner F, de Cid R, Gonzalez JR, Jarvis D, Heinrich J, Janson C, et al. Positionally cloned genes and age-specific effects in asthma and atopy: an international population-based cohort study (ECRHS). Thorax. 2010; 65:124-131.

Chae S-C Park Y-R, Oh G-J, Lee J-H, Chung H-T.The suggestive association of eotaxin-2 and eotaxin-3 gene polymorphisms in Korean population with allergic rhinitis. Immunogenetics. 2005; 56:760-764.

Chan IHS, Tang NLS, Leung TF, Ma SL, Zhang YP, Wong GWK, et al. Association of prostaglandin-endoperoxide synthase 2 gene polymorphisms with asthma and atopy in Chinese children. Allergy. 2007; 62:802-809.

Chan MA, Gigliotti NM, Meng J, Rosenwasser LJ. Asthma-related SNP in FCER2 is associated with increased expression of IL-4R on human B cells. International Journal of Immunogenetics. 2011; 38:533-538.

Chang Y, de Nadai P, Azzaoui I, Morales O, Delhem N, Vorng H, et al. The chemokine CCL18 generates adaptive regulatory T cells from memory CD4+ T cells of healthy but not allergic subjects. FASEB J. 2010; 24: 5063-5072.

Chatterjee R, Batra J, Ghosh B. A common exonic variant of interleukin21 confers susceptibility to atopic asthma. Int Arch Allergy Immunol. 2009; 148(2):137-46.

Chen R-F, Huang H-C, Ou C-Y, Hsu T-Y, Chuang H, Chang J-C, et al. MicroRNA-21 expression in neonatal blood associated with antenatal immunoglobulin E production and development of allergic rhinitis. Clin Exp Allergy. 2010;40:1482–90.

Cheong H -S, Kim LH, Park BL, Choi YH, Park H-S, Hong S-J, et al. Association analysis of interleukin 5 receptor alpha subunit (IL5RA) polymorphisms and asthma. J Hum Genet. 2005; 50:628-634.

Christensen U, Haagerup A, Binderup HG, Vestbo J, Kruse TA, Børglum AD. Family based association analysis of the IL2 and IL15 genes in allergic disorders. European Journal of Human Genetics. 2006; 14, 227-235.

Ciprandi G, Contini P, Murdaca G, DeAmici M, Gallina AM, Puppo F. Soluble HLA-G molecule in patients with perennial allergic rhinitis. Int Arch Allergy Immunol. 2009; 150(3):278-81.

Corry DB and Kheradmand F. Induction and regulation of the IgE response. Nature. 1999; 402 Supp; B18-B23.

Daniels S, Bhattacharrya S, James A, Leaves NI, Young A, Hill MR, et al. A genome-wide search for quantitative trait loci underlying asthma. Nature. 1996; 383:247-250.

Di Sciascio MB, Vianale G, Verna N, Petrarca C, Perrone A, Toniato E,et al. Eosinophil recruiting chemokines are down-regulated in peripheral blood mononuclear cells of allergic patients treated with deflazacort or desloratadine. Int J Immunopathol Pharmacol. 2007; 20(4):745-51.

Djidjik R, Ghaffor M, Brun M, Gharnaout M, Salah SS, Boukouaci W, et al. Constitutive nitric oxide synthase gene polymorphisms and house dust mite respiratory allergy in an Algerian patient group. Tissue Antigens. 2007; 71:160-164.

Eder W, Klimecki W, Yu L, von Mutius E, Riedler J, Braun-Fahrländer C, et al. Toll-like receptor 2 as a major gene for asthma in children of European farmers. J Allergy Clin Immunol. 2004; 113:482-8.

Eder W, limecki W, Yu L, von Mutius E, Riedler J, Braun-Fahrländer C, et al. Association between exposure to farming, allergies and genetic variation in CARD4/NOD1. Allergy. 2006; 61:1117-1124.

Eskandari HG, Unal M, Oztürk OG, Vayisoğlu Y, Muşlu N. Leukotriene C4 synthase A-444C gene polymorphism in patients with allergic rhinitis. Otolaryngol Head Neck Surg. 2006; 134:997–1000.

Fodor E, Kemény L, Koreck A, Széll M, Garaczi E, Polyánka H. The rs3761548 polymorphism of FOXP3 is a protective genetic factor against allergic rhinitis in the Hungarian female population. Human Immunology. 2011; 72:926-929.

Fryer AA, Spiteri MA, Bianco A, Hepple M, Jones PW, Strange RC, et al. The -403 G→A promoter polymorphism in the RANTES gene is associated with atopy and asthma. Genes and Immunity. 2000; 1:509-514.

Gao J, Li W, Willis-Owen SA, Jiang L, Ma Y, Tian X, et al. Polymorphisms of *PHF11* and *DPP10* Are Associated with Asthma and Related Traits in a Chinese Population. Respiration. 2010; 79:17-24.

Gao PS, Rafaels NM, Mu D, Hand T, Murray T, Boguniewicz M, et al. Genetic variants in thymic stromal lymphopoietin are associated with atopic dermatitis and eczema herpeticum. J Allergy Clin Immunol. 2010; 125:1403–1407

Genuneit J, Cantelmo JL, Weinmayr G, Wong GWK, Cooper PJ, Riikjärv M, et al. A multi-centre study of candidate genes for wheeze and allergy: the International Study of Asthma and Allergies in Childhood Phase 2. Clinical & Experimental Allergy. 2009; 39:1875-1888.

Gerbase MW, Keidel D, Imboden M, Gemperli a, Bircher a, Schmid-Grendelmeier P, et al. Effect modification of immunoglobulin E-mediated atopy and rhinitis by glutathione S-transferase genotypes in passive smokers. Clinical & Experimental Allergy. 2011; 41:1579-1586.

Gilliland FD, Li YF, Saxon A, Diaz-Sanchez D. Effect of glutathione-S-transferase M1 and P1 genotypes on xenobiotic enhancement of allergic responses: randomised, placebo-controlled crossover study. Lancet. 2004 Jan; 363(9403):119-25.

Giubergia V, Zelazko M, Roy A, Gravina LP, Pena HG, Chertkoff L.. _ᵝ2_-Adrenergic polymorphisms and totalserum IgE levels in children with asthma from Argentina. Ann Allergy Asthma Immunol. 2009; 102:308-313.

Granada M, Wilk JB, Tuzova M, Strachan DP, Weidinger S, Albrecht E, et al. A genome-wide association study of plasma total IgE concentrations in the Framingham Heart Study. J Allergy Clin Immunol. 2012; 129(3):840-845.e21.

Gudbjartsson DF, Bjornsdottir US, Halapi E, Helgadottir A, Sulem P, Jonsdottir GM,et al. Sequence variants affecting eosinophil numbers associate with asthma and myocardial infarction. Nat Genet. 2009; 41(3):342-7.

Han D, She W, Zhang L. Association of the CD14 gene polymorphism C-159T with allergic rhinitis. Am J Rhinol Allergy. 2010; 24(1):e1-3.

Hancock DB, Romieu I, Shi M, Sienra-Monge J-J, Wu H, Chiu GY, et al. Genome-Wide Association Study Implicates Chromosome 9q21.31 as a Susceptibility Locus for Asthma in Mexican Children. PLoS genet. 2009; 5(8): e1000623.

Harada M, Hirota T, Jodo AI, Hitomi Y, Sakashita M, Tsunoda T, et al. Thymic Stromal Lymphopoietin Gene Promoter Polymorphisms Are Associated with Susceptibility to Bronchial Asthma. Am J Respir Cell Mol Biol. 2011; 44: 787-793.

Hasegawa K, Tamari M, Shao C, Shimizu M, Takahashi N, Mao X-Q, et al. Variations in the C3, C3a receptor, and C5 genes affect susceptibility to bronchial asthma. Hum Genet. 2004; 115:295-301.

Hatsushika K, Hirota T, Harada M, Sakashita M, Kanzaki M, Takano S, et al. Transforming growth factor-beta(2) polymorphisms are associated with childhood atopic asthma. Clin Exp Allergy. 2007 Aug;37(8):1165-74.

Hattori H, Rosas LE, Okano M, Durbin JE, Nishizaki K, Satoskar AR. STAT1 is involved in the pathogenesis of murine allergic rhinitis. Am J Rhinol. 2007 Mar-Apr;21(2):241-7.

Hecker M, Bohnert A, König IR, Bein G, Hackstein H. Novel genetic variation of human interleukin-21 receptor is associated with elevated IgE levels in females. Genes Immun. 2003 Apr;4(3):228-33.

Hersberger M, Thun G-A, Imboden M, Brandstätter A, Waechter V, Summerer M, et al. Association of STR polymorphisms in *CMA1* and *IL-4* with asthma and atopy: The SAPALDIA Cohort. Human Immunology 2010; 71:1154-1160.

Himes BE, Hunninghake GM, Baurley JW, Rafaels NM, Sleiman P, Strachan DP, et al. Genome-wide Association Analysis Identifies PDE4D as an Asthma-Susceptibility Gene. Am J Hum Genet. 2009; 84:581–93.

Hiromura Y, Kishida T, Nakano H, Hama T, Imanishi J, Hisa Y, et al. IL-21 administration into the nostril alleviates murine allergic rhinitis. J Immunol. 2007; 179(10):7157-65.

Hollá LI, Buckova D, Kuhrova V, Stejskalova A, Francova H, Znojil V,et al. Prevalance of endothelial nitric oxide synthase gene polymorphisms in patients with atopic asthma. Clin Exp Allergy 2002; 32:1193-1198.

Hollá LI Schüller M, Bucková D, Vácha J. Neuronal nitric oxide synthase gene polymorphism and IgE-mediated allergy in the Central European population. Allergy 2004: 59:548-552.

Hollá LI, Stejskalova A, Znojil V, Vasku A. Analysis of the inducible nitric oxide synthase gene polymorphisms in Czech patients with atopic diseases. Clinical and Experimental Allergy. 2006; 36:1592-1601.

Hon KL, Ching GK, Ng PC, Leung TF. Exploring CCL18, eczema severity and atopy. Pediatr Allergy Immunol. 2011; 22:704–707.

Hrdlickova B and Izakovicova-Holla L. Association of Single Nucleotide Polymorphisms in the Eosinophil Peroxidase Gene with Allergic Rhinitis in the Czech Population. Int Arch Allergy Immunol. 2009; 150:184-191.

Hsi, L, Kundu S, Palomo J, Xu B, Ficco R, Vogelbaum MA,et al. Silencing IL-13Rα2 Promotes Glioblastoma Cell Death via Endogenous Signaling. Mol. Cancer Ther. 2011; 10(7): 1149–60.

Hubiche T, Ged C, Benard A, Léauté-Labrèze C, McElreavey K, de Verneuil H, et al. Analysis of *SPINK 5, KLK 7* and *FLG* Genotypes in a French Atopic Dermatitis Cohort. Acta Derm Venereol. 2007; 87:499-505.

Husemoen LLN, Toft U, Fenger M, Jørgensen T, Johansen N, Linneberg A.The association between atopy and factors influencing folate metabolism: is low folate status causally related to the development of atopy? International Journal of Epidemiology. 2006; 35:954-961.

Hussein YM, Ahmad a S, Ibrahem MM, El Tarhouny SA, Shalaby SM, Elshal AS,Interferon Gamma Gene Polymorphism as a Biochemical Marker in Egyptian Atopic Patients. J Investig Allergol Clin Immunol 2009; 19(4):292-298.

Hussein YM, Shalaby SM, Mohamed RH, Hassan TH. Association between genes encoding components of the IL-10/IL-0 receptor pathway and asthma in children. Ann Allergy Asthma Immunol. 2011; 106:474-480.

Imboden M, Nieters a, Bircher a J, Brutsche M, Becker N, Wjst M, et al. Cytokine gene polymorphisms and atopic disease in two European cohorts. (ECRHS-Basel and SAPALDIA). Clinical and Molecular Allergy. 2006; 4:9. doi:10.1186/1476-7961-4-9

Immervol T, Loesgen S, Dütsch G, Gohlke H, Herbon N, Klugbauer S, et al. Fine Mapping and Single Nucleotide Polymorphism Association Results of Candidate Genes for Asthma and Related Phenotypes. Human Mutation. 2001; 18:327-336.

Inoue H, Mashimo Y, Funamizu M, Shimojo N, Hasegawa K, Hirota T, et al. Association study of the C3 gene with adult and childhood asthma. J Hum Genet. 2008; 53:728-738.

Isidoro-García M, Sanz C, García-Solaesa V, Pascual M, Pescador DB, Lorente F, et al. *PTGDR* gene in asthma: a functional, genetic, and epigenetic study. Allergy 2011; 66:1553-1562.

Ismaïl A, Bousaffara R, Kaziz J, Zili J, el Kamel A, Tahar Sfar M, et al. Polymorphism in transporter antigen peptides gene (TAP1) associated with atopy in Tunisians. J Allergy Clin Immunol. 1997; 99:216-23.

Jang N, Stewart G, Jones G. Polymorphisms within the PHF11 gene at chromosome 13q14 are associated with childhood atopic dermatitis. Genes and Immunity. 2005; 6:262-264.

Johnson LG, Schwartz SM, Malkki M, Du Q, Petersdorf EW, Galloway DA, et al. Risk of Cervical Cancer Associated with Allergies and Polymorphisms in Genes in the Chromosome 5 Cytokine Cluster. Cancer Epidemiol Biomarkers Prev. 2011; 20(1): 199-207.

Joki-Erkkilä V-P, Karjalainen J, Hulkkonen J, Pessi T, Nieminen MM, Aromaa A, et al. Allergic rhinitis and polymorphisms of the interleukin 1 gene complex. Ann Allergy Asthma Immunol. 2003; 91:275–279.

Jönsson, Håkansson LD, Jõgi R, Janson C, Venge P. Associations of ECP (eosinophil cationic protein)-gene polymorphisms to allergy, asthma, smoke habits and lung function in two Estonian and Swedish sub cohorts of the ECRHS II study. BMC Pulmonary Medicine. 2010; 10:36. doi:10.1186/1471-2466-10-36

Kabesch M, Depner M, Dahmen I, Weiland SK, Vogelberg C, Niggemann B, et al. Polymorphisms in eosinophil pathway genes, asthma and atopy. Allergy. 2007; 62: 423–428.

Kalpaklioğlu AF and Turan M. Possible association between cockroach allergy and HLA class II antigens. Ann Allergy Asthma Immunol. 2002; 89:155-158.

Kang I, An X, Oh Y-K, Lee SH, Jung HM, Chae S-C, et al. Identification of polymorphisms in the RNase3 gene and the association with allergic rhinitis. Eur Arch Otorhinolaryngol. 2010; 267:391-395.

Karjalainen J, Hulkkonen J, Pessi T, Huhtala H, Nieminen MM, Aromaa A, et al. The *IL1A* genotype associates with atopy in nonasthmatic adults. J Allergy Clin Immunol. 2002; 110:429-434.

Kim JJ, Lee JH, Jang CH, Kim YS, Chae SC, Chung HT, et al. Chemokine RANTES Promoter Polymorphisms in Allergic Rhinitis. Laryngoscope. 2004; 114:666–669.

Kim KR, Cho SH, Choi SJ, Jeong JH, Lee SH, Park CW, et al. *TAP1* and *TAP2* Gene Polymorphisms in Korean Patients with Allergic Rhinitis. J Korean Med Sci. 2007; 22:825-831.

Kobayashi S, Haruo N, Sugane K, Ochs HD, Agematsu K. Interleukin-21 stimulates B-cell immunoglobulin E synthesis in human beings concomitantly with activation-induced cytidine deaminase expression and differentiation into plasma cells. Hum Immunol. 2009; 70(1):35-40.

Kondo N, Matsui E, Kaneko H, Fukao T, Teramoto T, Inoue R, et al. Reduced interferon-gamma production and mutations of the interleukin-12 receptor beta-2 chain gene in atopic subjects. Int Arch Allergy Immunol. 2001;124:117–20.

Konno S, Hizawa N, Yamaguchi E, Jinushi E, Nishimura M.(CCTTT)_n_ repeat polymorphism in the NOS2 gene promoter is associated with atopy. J Allergy Clin Immunol. 2001; 108:810-814.

Kormann MSD, Ferstl R, Depner M, Klopp N, Spiller S, Illig T, et al. Rare TLR2 mutations reduce TLR2 receptor function and can increase atopy risk. Allergy. 2009; 64:636-642.

Krasznai M, Szaniszlo K, Kraxner H, Vargha E, Kovacs M, Borocz Z, et al. Association of TLR-4 and TNF-alpha polymorphisms with clinical symptoms and cytokine levels in patients with allergic rhinitis. Eur Arch Otorhinolaryngol. 2011; 268:561-567.

Kruse S, Kuehr J, Moseler M, Kopp M V, Kurz T, Deichmann KA, et al. Polymorphisms in the *IL18* gene are associated with specific sensitization to common allergens and allergic rhinitis. J Allergy Clin Immunol. 2003; 111:117-122.

Ku M-S, Sun H-L, Lu K-H, Sheu J-N, Lee H-S, Yang S-F, et al. The *CC16* A38G polymorphism is associated with the development of asthma in children with allergic rhinitis. Clinical & Experimental Allergy. 2011; 41:794-800.

Kwon J-W, Kim T-W, Cho S-H, Min K-U, Park H-W. Serum YKL-40 levels are correlated with symptom severity in patients with allergic rhinitis. Allergy. 2011; 66:1252-1259.

Lee HJ, Ha SJ, Han H, Kim JW.Distribution of HLA-A, B alleles and polymorphisms of TAP and LMP genes in Korean patients with atopic dermatitis. Clinical and Experimental Allergy. 2001; 31:1867-1874.

Leung TF, Tang NLS, Lam CWK, Li AM, Chan IHS, Ha G.Thromboxane A2 receptor gene polymorphism is associated with the serum concentration of cat-specific immunoglobulin E as well as the development and severity of asthma in Chinese children. Pediatri Allergy Immunol. 2002; 13: 10-17.

Leung TF, Tang NLS, Lam CWK, Li a M, Fung SLM, Chan IHS, et al. *RANTES* G-401A polymorphism is associated with allergen sensitization and FEV_1_ in Chinese children. Respiratory Medicine. 2005; 99:216-219.

Li CS, Chae SC, Lee JH, Zhang Q, Chung HT. Identification of single nucleotide polymorphisms in FOXJ1 and their association with allergic rhinitis. J Hum Genet. 2006; 51(4):292-297.

Li H, Romieu I, Wu H, Sienra-Monge J-J, Ramírez-Aguilar M, del Río-Navarro BE, et al. Genetic polymorphisms in transforming growth factor beta-1 (*TGFB1*) and childhood asthma and atopy. J Hum Genet. 2007; 121(5):529-538.

Li X, Howard TD, Zheng SL, Haselkorn T, Peters SP, Meyers DA, et al. Genome-wide association study of asthma identifies RAD50-IL13 and HLA-DR/DQ regions. J Allergy Clin Immunol 2010; 125:328-335.

Li Y, Guo B, Zhang L, Han J, Wu B, Xiong H.Association between C-589T polymorphisms of interleukin-4 gene promoter and asthma: A meta-analysis. Respiratory Medicine. 2008; 102:984-992.

Litonjua AA, Belanger K, Celedón JC, Milton DK, Bracken MB, Kraft P, et al. Polymorphisms in the 5’ region of the CD14 gene are associated with eczema in young children. J Allergy Clin Immunol. 2005; 115:1056-1062.

Lu M-P, Chen RX, Wang ML, Zhu XJ, Zhu L-P, Yin M et al. Association Study on *IL4*, *IL13* and *IL4RA* Polymorphisms in Mite-Sensitized Persistent Allergic Rhinitis in a Chinese Population. PLoS ONE. 2011; 6(11): e27363. doi:10.1371/journal.pone.0027363

Martino DJ, Bosco A, McKenna KL, Hollams E, Mok D, Holt PG, Prescott SL. T-cell activation genes differentially expressed at birth in CD4(+) T-cells from children who develop IgE food allergy. Allergy. 2012; 67(2):191-200.

Mathias, RA, Grant A V., Rafaels N, Hand T, Gao L, Vergara C, et al. A Genome-Wide Association Study on African-Ancestry Populations for Asthma. J Allergy Clin Immunol. 2010; 125(2): 336–346.e4.

McIntire JJ, Umetsu DT, DeKruyff RH. TIM-1, a novel allergy and asthma susceptibility gene. Springer Semin Immunopathol. 2004; 25(3-4):335-48.

Meng J, Thongngarm T, Nakajima M, Yamashita N, Ohta K, Bates CA, et al. Association of Transforming Growth Factor-β1 Single Nucleotide Polymorphism C-509T with Allergy and Immunological Activities. Int Arch Allergy Immunol. 2005; 138:151-160.

Micheal S, Minhas K, Ishaque M, Ahmed F, Ahmed A.. Promoter Polymorphisms of the *CD14* Gene Are Associated With Atopy in Pakistani Adults. J Investig Allergol Clin Immunol. 2011; 21(5):394-397.

Michel S, Liang L, Depner M, Klopp N, Ruether A, Kumar A, et al. Unifying Candidate Gene and GWAS Approaches in Asthma. PLoS ONE. 2010; 5(11): e13894. doi:10.1371/journal.pone.0013894

Miedema KGE, Tissing WJE, Te Poele EM, Kamps WA, Alizadeh BZ, Kerkhof M, et al. Polymorphisms in the TLR6 gene associated with the inverse association between childhood acute lymphoblastic leukemia and atopic disease. Leukemia. 2011; 1-8. doi:10.1038/leu.2011.341

Minelli C, Granell R, Newson R, Rose-Zerilli MJ, Torrent M, Ring SM, et al. Glutathione-S-transferase genes and asthma phenotypes: a Human Genome Epidemiology (HuGE) systematic review and meta-analysis including unpublished data. International Journal of Epidemiology. 2010; 39:539-562. doi:10.1093/ije/dyp337

Minhas K, Micheal S, Ahmed F, Ahmed A. Strong Association Between the -308 TNF Promoter Polymorphism and Allergic Rhinitis in Pakistani Patients. J Investig Allergol Clin Immunol. 2010; 20(7):563-566.

Mobini R, Andersson BA, Erjefält J, Hahn-Zoric M, Langston MA, Perkins AD, et al. A module-based analytical strategy to identify novel disease-associated genes shows an inhibitory role for interleukin 7 Receptor in allergic inflammation. BMC Syst Biol. 2009; 3:19.

Moffatt MF, Gut IG, Demenais F, Strachan DP, Bouzigon E, Heath S, et al. A Large-Scale, Consortium-Based Genomewide Association Study of Asthma. N Engl J Med. 2010; 363:1211-1221.

Moissidis I, Chinoy B, Yanamandra K, Napper D, Thurmon T, Bocchini J, et al. Association of IL-13, RANTES, and leukotriene C4 synthase gene promoter polymorphisms with asthma and/or atopy in African Americans. Genet Med. 2005: 7(6):406-410.

Mostecki J, Cassel SL, Klimecki WT, Stern DA, Knisz J, Iwashita S,et al. A SOCS-1 promoter variant is associated with total serum IgE levels. J Immunol. 2011 Sep 1;187(5):2794-802.

Mou Z, Shi J, Tan Y, Xu R, Zhao Z, Xu G, et al. Association between *TIM-1* Gene Polymorphisms and Allergic Rhinitis in a Han Chinese Population. J Investig Allergol Clin Immunol. 2010; 20(1):3-8.

Muller B, de Groot EJ, Kortekaas IJ, Fokkens WJ, van Drunen CM. et al. Nasal endothelial interleukin-10 expression is negatively correlated with nasal symptoms after allergen provocation. Allergy. 2009; 64(5):738-45.

Munthe-Kaas MC, Carlsen KH, Helms PJ, Gerritsen J, Whyte M, Feijen M, et al. *CTLA-4* polymorphisms in allergy and asthma and the T_H_1/T_H_2 paradigm. J Allergy Clin Immunol. 2004;114:280-287.

Munthe-Kaas MC, Carlsen KL, Carlsen KH, Egeland T, Håland G, Devulapalli CS, et al. HLA Dr-Dq haplotypes and the TNFA-308 polymorphism: associations with asthma and allergy. Allergy 2007: 62: 991–998

Murk W, Walsh K, Hsu L-I, Zhao L, Bracken MB, Dewan AT. Attempted Replication of 50 Reported Asthma Risk Genes Identifies a SNP in RAD50 as Associated with Childhood Atopic Asthma. Hum Hered 2011; b71:97-105.

Nagata H, Mutoh H, Kumahara K, Arimoto Y, Tomemori T, Sakurai D, et al. Association between nasal allergy and a coding variant of the *Fc****ε****RI*β gene Glu237Gly in a Japanese population. Hum Genet. 2001; 109:262-266.

Naito T, Tanaka H, Naoe Y, Taniuchi I.. Transcriptional control of T-cell development. International Immunology. 2011; 23(11):661-668.

Nakamura H, Miyagawa K, Ogino K, Endo T, Imai T, Ozasa K, et al. High contribution contrast between the genes of eosinophil peroxidase and IL-4 receptor α-chain in Japanese cedar pollinosis. J Allergy Clin Immunol. 2003; 112:1127-31.

Nakamura H, Higashikawa F, Nobukuni Y, Miyagawa K, Endo T, Imai T, et al. Genotypes and haplotypes of CCR2 and CCR3 genes in Japanese cedar pollinosis. Int Arch Allergy Immunol. 2007;142(4):329-34.

Nakao F, Ihara K, Kusuhara K, Sasaki Y, Kinukawa N, Takabayashi A, et al. Association of IFN-γ and IFN regulatory factor 1 polymorphisms with childhood atopic asthma. J Allergy Clin Immunol. 2001; 107:499-504.

Namkung JH, Lee JE, Kim E, Cho HJ, Kim S, Shin ES, et al. IL-5 and IL-5 receptor alpha polymorphisms are associated with atopic dermatitis in Koreans. Allergy 2007; 62:934-942.

Nieters A, Linseisen J, Becker N. Association of polymorphisms in Th1, Th2 cytokine genes with hayfever and atopy in a subsample of EPIC-Heidelberg. Clin Exp Allergy. 2004; 34:346-353.

Ober C and Hoffjan S. Asthma genetics 2006: the long and winding road to gene discovery. Genes and Immunity. 2006; 7: 95-100.

Ober C and Yao T-C. The genetics of asthma and allergic disease: a 21st century perspective. Immunological Reviews. 2011; 242:10-30.

Oh DY, Schumann RR, Hamann L, Neumann K, Worm M, Heine G. et al. Association of the *toll-like receptor* 2 A-16934T promoter polymorphism with severe atopic dermatitis. Allergy. 2009; 64:16080-1615.

Owczarek W, Paplińska M, Targowski T, Jahnz-Różyk K, Paluchowska E, Kucharczyk A, et al. Analysis of eotaxin 1/CCL11, eotaxin 2/CCL24 and eotaxin 3/CCL26 expression in lesional and non-lesional skin of patients with atopic dermatitis. Cytokine. 2010; 50(2):181-5.

Pawankar R, Okuda M, Yssel H, Okumura K, and Ra C. Nasal Mast Cells in Perennial Allergic Rhinitics Exhibit Increased Expression of the Fc epsilonRI, CD40L, IL-4, and IL-13, and Can Induce IgE Synthesis in B Cells. J Clin Invest. 1997; 99(7): 1492–99.

Pawankar R, Mori S, Ozu C, Kimura S. Overview on the pathomechanisms of allergic rhinitis. Asia Pac Allergy. 2011; 1:157–67.

Pène J, Guglielmi L, Gauchat JF, Harrer N, Woisetschläger M, Boulay V,et al. IFN-gamma-mediated inhibition of human IgE synthesis by IL-21 is associated with a polymorphism in the IL-21R gene. J Immunol. 2006; 177(8):5006-13.

Pinto LA, Steudemann L, Depner M, Klopp N, Illig T, Weiland SK, et al. *STAT1* gene variations, IgE regulation and atopy. Allergy 2007: 62: 1456–1461.

Ponińska J, Samoliński B, Tomaszewska A, Raciborski F, Samel-Kowalik P, et al. Filaggrin Gene Defects are Independent Risk Factors for Atopic Asthma in a Polish Population: A Study in ECAP Cohort. PLoS ONE. 2011; 6(2):e16933. doi:10.1371/journal.pone.0016933

Poon AH, Laprise C, Lemire M, Montpetit A, Sinnett D, Schurr E, et al. Association of Vitamin D Receptor Genetic Variants with Susceptibility to Asthma and Atopy. Am J Respir Crit Care Med. 2004; 170:967-973.

Potaczek DP, Sanak M, Szczeklik A. Additive association between *FCER1A* and *FCER1B* genetic polymorphisms and total serum IgE levels. Allergy. 2007; 62:1095-1099.

Purwar R, Langer K, Werfel T. Polymorphisms within the C3 gene are associated with specific IgE levels to common allergens and super-antigens among atopic dermatitis patients. Experimental Dermatology. 2009; 18:30-34.

Qian F-H, Zhang Q, Zhou LF, Jin GF, Bai JL, Yin KS. Polymorphisms in the *Toll-like Receptor 2* Subfamily and Risk of Asthma: A Case-control Analysis in a Chinese Population. J Investig Allergol Clin Immunol. 2010; 20(4):340-346.

Raby BA, Lazarus R, Silverman EK, Lake S, Lange C, Wjst M, et al. Association of Vitamin D Receptor Gene Polymorphisms with Childhood and Adult Asthma. Am J Respir Crit Care Med. 2004; 170:1057-1065.

Ramasamy A, Curjuric I, Coin LJ, Kumar A, McArdle WL, Imboden M, et al. A genome-wide meta-analysis of genetic variants associated with allergic rhinitis and grass sensitization and their interaction with birth order. J Allergy Clin Immunol. 2011; 128:996-1005.

Reddy P, Naidoo RN, Robins TG, Mentz G, London SJ, Li H, et al. GSTM1, GSTP1, and NQO1 Polymorphisms and Susceptibility to Atopy and Airway Hyperresponsiveness among South African Schoolchildren. Lung. 2010; 188:409-414.

Reijmerink NE, Bottema RW, Kerkhof M, Gerritsen J, Stelma FF, Thijs C,et al. TLR-related pathway analysis: novel gene-gene interactions in the development of asthma and atopy. Allergy. 2010 Feb;65(2):199-207.

Ricci G, Astolfi A, Remondini D, Cipriani F, Formica S, Dondi A,et al. Pooled Genome-Wide Analysis to Identify Novel Risk Loci for Pediatric Allergic Asthma. PLoS ONE. 2011; 6(2): e16912. doi:10.1371/journal.pone.0016912

Rothenberg ME, Wen T, Shik D, Cole ET, Mingler MM, Munitz A. IL-13 Receptor α1 Differentially Regulates Aeroallergen-Induced Lung Responses. The Journal of Immunology. 2011; 187:4873-4880.

Sääf AM, Tengvall-Linder M, Chang HY, Adler AS, Wahlgren C-F, Scheynius A, et al. Global expression profiling in atopic eczema reveals reciprocal expression of inflammatory and lipid genes. PLoS One. 2008; 3:e4017.

Sakashita M, Yoshimoto T, Hirota T, Harada M, Okubo K, Osawa Y, et al. Association of serum interleukin-33 level and the interleukin-33 genetic variant with Japanese cedar pollinosis. Clinical and Experimental Allergy. 2008; 38:1875-1881.

Salib RJ, Kumar S, Wilson SJ, Howarth PH. Nasal mucosal immunoexpression of the mast cell chemoattractants TGF-beta, eotaxin, and stem cell factor and their receptors in allergic rhinitis. J Allergy Clin Immunol. 2004;114(4):799-806.

Salib, RJ. Transforming Growth Factor-Beta Gene Expression Studies in Nasal Mucosal Biopsies in Naturally Occurring Allergic Rhinitis. Ann R Coll Surg Engl. 2007; 89(6): 563–73.

Sanz C, Isidoro-García M, Dávila I, Moreno E, Laffond E, Avila C, et al. Promoter genetic variants of prostanoid DP receptor (PTGDR) gene in patients with asthma. Allergy. 2006; 61: 543-548.

Schuttelaar MLA, Kerkhof M, Jonkman MF, Koppelman GH, Brunekreef B, de Jongste JC, et al. Filaggrin mutations in the onset of eczema, sensitization, asthma, hay fever and the interaction with cat exposure. Allergy. 2009; 64:1758-1765.

Senthilselvan A, Rennie D, Chénard L, Burch LH, Babiuk L, Schwartz DA, et al. Association of polymorphisms of toll-like receptor 4 with a reduced prevalence of hay fever and atopy. Ann Allergy Asthma Immunol. 2008; 100:463-468.

Sharma S, Rajan UM, Kumar A, Soni A, Ghosh B. A novel (TG)_n_ (GA)_m_ repeat polymorphism 254 bp downstream of the mast cell chymase (*CMA1*) gene is associated with atopic asthma and total serum IgE levels. Hum Genet. 2005; 50:276-282.

Sharma S, Sharma A, Kumar S, Sharma SK, Ghosh B.et al. Association of *TNF* Haplotypes with Asthma, Serum IgE Levels, and Correlation with Serum TNF-α Levels. Am J Respir Cell Mol Biol. 2006; 35:488-495.

Shi J, Misso NL, Kedda M, Horn J, Welch MD, Duffy DL, et al. Cyclooxygenase-2 gene polymorphisms in an Australian population: association of the ̶ 1195G > A promoter polymorphism with mild asthma. Clinical and Experimental Allergy. 2008; 38:913-920.

Shilling RA, Pinto JM, Decker DC, Schneider DH, Bandukwala HS, Schneider JR, et al. Cutting Edge: Polymorphisms in the *ICOS* Promoter Region Are Associated with Allergic Sensitization and Th2 Cytokine Production. The Journal of Immunology. 2005; 175:2061-2065.

Shimizu M, Matsuda A, Yanagisawa K, Hirota T, Akahoshi M, Inomata N,et al. Functional SNPs in the distal promoter of the ST2 gene are associated with atopic dermatitis. Hum Mol Genet. 2005 Oct 1;14(19):2919-27.

Shin, H-D, Park BL, Jung JH, Wang HJ, Park HS, Choi BW, et al. Association of thromboxane A2 receptor (*TBXA2R*) with atopy and asthma. J Allergy Clin Immunol. 2003; 112(2):454-456.

Shin HD, Kim LH, Park BL, Jung JH, Kim JY, Chung I-Y, et al. Association of Eotaxin gene family with asthma and serum total IgE. Human Molecular Genetics. 2003; 12(11):1279-1285.

Shin, EK, Lee S-H, Cho S-H, Jung S, Yoon SH, Park SW, et al. Association between colony-stimulating factor 1 receptor gene polymorphisms and asthma risk. Hum Genet. 2010; 128:293-302.

Shirasaki H, Kikuchi M, Kanaizumi E, Himi T. Accumulation of CRTH2-positive leukocytes in human allergic nasal mucosa. Ann Allergy, Asthma Immunol. 2009;102(2):110-5.

Shirasaki H, Kanaizumi E, Seki N, Himi T. Localization and upregulation of the nasal histamine H1 receptor in perennial allergic rhinitis. Mediators Inflamm. 2012; 2012:951316.

Sleiman, PMA, Flory J, Imielinski M, Bradfield JP, Annaiah K, Willis-Owen SAG, et al. Variants of DENND1B Associated with Asthma in Children. N Engl. J Med. 2010; 362(1): 36–44.

Sohn MH, Lee JH, Kim KW, Kim SW, Lee SH, Kim KE,et al. Genetic variation in the promoter region of chitinase 3-like 1 is associated with atopy. Am J Respir Crit Care Med. 2009; 179(6):449-56.

Souwer Y, Szegedi K, Kapsenberg ML, de Jong EC. IL-17 and IL-22 in atopic allergic disease. Current Opinion in Immunology. 2010; 22:821-826.

Su, D, Zhang X, Sui H, Lü F, Jin L, Zhang J. Association of ADAM33 gene polymorphisms with adult allergic asthma and rhinitis in a Chinese Han population. BMC Med Genet. 2008; 9:82. doi:10.1186/1471-2350-9-82.

Suttner K, Depner M, Wetzke M, Klopp N, von Mutius E, Illig T, et al. Genetic variants harbored in the forkhead box protein 3 locus increase hay fever risk. J Allergy Clin Immunol. 2010; 125(6):1395-1399.

Takizawa R, Pawankar R, Yamagishi S, Takenaka H, Yagi T.et al. Increased expression of HLA-DR and CD86 in nasal epithelial cells in allergic rhinitics: antigen presentation to T cells and up-regulation by diesel exhaust particles. Clinical and Experimental Allergy. 2007; 37: 420-433.

Tantisira KG, Silverman ES, Mariani TJ, Xu J, Richter BG, Klanderman BJ, et al. *FCER2*: A pharmacogenetic basis for severe exacerbations in children with asthma. J Allergy Clin Immunol. 2007; 120:1285-91.

Thuesen BH, Husemoen LLN, Ovesen L, Jørgensen T, Fenger M, Gilderson G, et al. Atopy, asthma, and lung function in relation to folate and vitamin B12 in adults. Allergy. 2010; 65:1446-1454.

Torgerson, DG, Ampleford EJ, Chiu GY, Gauderman WJ, Gignoux CR, Graves PE et al. Meta-Analysis of Genome-Wide Association Studies of Asthma in Ethnically Diverse North American Populations. 2011; Nature genetics 43(9): 887–92.

Trabetti E, Patuzzo C, Malerba G, Galavotti R, Martinati LC, Boner AL, et al. Association of a lymphotoxin α gene polymorphism and atopy in Italian families. J Med Genet. 1999; 36:323-325.

Van den Oord, RAHM, Sheikh A. Filaggrin gene defects and risk of developing allergic sensitisation and allergic disorders: systematic review and meta-analysis. BMJ 2009; 339:b2433. doi:10.1136/bmj.b2433

Walley AJ, Chavanas S, Moffatt MF, Esnouf RM, Ubhi B, Lawrence R, et al. Gene polymorphism in Netherton and common atopic disease. Nature Genetics. Oct 2001; 29:175-178.

Wan YI, Strachan DP, Evans DM, Henderson J, McKeever T, Holloway JW, et al. A genome-wide association study to identify genetic determinants of atopy in subjects from the United Kingdom. J Allergy Clin Immunol. 2011; 127:223-231.

Wang, M, Xing Z-M, Yu D-L, Yan Z, Yu L-S. Association between HLA class II locus and the susceptibility to *Artemisia* pollen–induced allergic rhinitis in Chinese population. Otolaryngol Head Neck Surg. 2004; 130:192-196.

Wang TN, Chiang W, Tseng HI, Chu YT, Chen WY, Shih NH, et al. The polymorphisms of Eotaxin 1 and CCR3 genes influence on serum IgE, Eotaxin levels and mild asthmatic children in Taiwan. Allergy. 2007; 62:1125-1130.

Wang Y-H, Liu Y-J. Thymic stromal lymphopoietin, OX40-ligand, and interleukin-25 in allergic responses. Clinical & Experimental Allergy. 2009; 39: 798-806.

Weidinger S, Rümmler L, Klopp N, Wagenpfeil S, Baurecht HJ, Fischer G, et al. Association study of mast cell chymase polymorphisms with atopy. Allergy. 2005A; 60:1256-1261.

Weidinger S, Klopp N, Rummler L, Wagenpfeil S, Novak N, Baurecht H-J, et al. Association of *NOD1* polymorphisms with atopic eczema and related phenotypes. J Allergy Clin Immunol. 2005B; 116:177-184.

Weidinger S, Gieger C, Rodriguez E, Baurecht H, Mempel M, Klopp N,et al. Genome-Wide Scan on Total Serum IgE Levels Identifies FCER1A as Novel Susceptibility Locus. PLoS Genet. 2008; 4(8): e1000166. doi:10.1371/journal.pgen.1000166

Williams, CMM, Rahman S, Hubeau C, Ma H-L. Cytokine Pathways in Allergic Disease. Toxicol Pathol 1 February 2012. Published online. DOI: 10.1177/0192623311430694

Wisniewski JA, Borish L. Novel cytokines and cytokine-producing T cells in allergic disorders. Allergy Asthma Proc. 2011; 32: 83-94.

Woszczek G, Borowiec M, Ptasinska a, Kosinski S, Pawliczak R, Kowalski ML.β_2_-ADR haplotypes/polymorphisms associate with bronchodilator response and total IgE in grass allergy. Allergy. 2005; 60:1412-1417.

Yang KD, Liu C, Chang J-C, Chuang H, Ou C-Y, Hsu T-Y, et al. Polymorphism of the immune-braking gene CTLA-4 (149) involved in gender discrepancy of serum total IgE levels and allergic diseases. Clin Exp Allergy. 2004; 34:32-37.

Yokoe T, Suzuki N, Minoguchi K, Adachi M, Sakane T. Analysis of IL-12 receptor beta-2 chain expression of circulating T lymphocytes in patients with atopic asthma. Cell Immunol 2001;208:34–42.

Yu J, Kang I-H, Chun S-W, Yun K-J, Moon H-B, Chae S-C. Identifying the polymorphisms in the thymic stromal lymphopoietin receptor (TSLPR) and their association with asthma. BMB Rep. 2010; 43:499–505.

Zeyrek D, Demir E, Alpman A, Ozkinay F, Gulen F, Tanac R. Association of interleukin-1beta and interleukin-1 receptor antagonist gene polymorphisms in Turkish children with atopic asthma. Allergy Asthma Proc. 2008; 29:468 -474.

Zhang H, Yang P, Zhou H, Meng Q, Huang X. Involvement of Foxp3-expressing CD4+ CD25+ regulatory T cells in the development of tolerance induced by transforming growth factor-beta2-treated antigen-presenting cells. Immunology. 2008; 124(3):304-14.

Zhang, L, Zhang Y, Desrosiers M, Wang C, Zhao Y, Han D.Genetic association study of *FOXP3* polymorphisms in allergic rhinitis in a Chinese population. Human Immunology. 2009A; 70:930-934.

Zhang, L, Wang X, Han D, Duan H, Zhao Y.Association of single nucleotide polymorphisms in GATA-3 with allergic rhinitis. Acta Oto-Laryngologica. 2009B; 129:190-194.

Zhang, Y, Leaves NI, Anderson GG, Ponting CP, Broxholme J, Holt R, et al.. Positional cloning of a quantitative trait locus on chromosome 13q14 that influences immunoglobulin E levels and asthma. Nature Genetics. 2003; 34(2):181-186.

Zhou, H, Hong X, Jiang S, Dong H, Xu X, Xu X.Analyses of associations between three positionally cloned asthma candidate genes and asthma or asthma-related phenotypes in a Chinese population. BMC Medical Genetics. 2009; 10:123. doi:10.1186/1471-2350-10-123

Zhou, J, Zhou Y, Lin L, Wang J, Peng X, Li J,et al. Association of polymorphisms in the promoter region of FCER1A gene with atopic dermatitis, chronic uticaria, asthma, and serum immunoglobulin E levels in a Han Chinese population. Hum Immunol. 2012; 73(3):301-305. doi:10.1016/j.humimm.2011.12.001

Zhu G, Carlsen K, Carlsen K-H, Lenney W, Silverman M, Whyte MK, et al. Polymorphisms in the endothelin-1 (EDN1) are associated with asthma in two populations. Genes Immun. 2008;9:23–9. 10.1038/sj.gene.6364441
