# Supplementary material for: Association between Variants in Atopy-Related Immunologic Candidate Genes and Pancreatic Cancer Risk
Source: PLoS One. 2015 May 6;10(5):e0125273. doi: 10.1371/journal.pone.0125273 (PMC4422524; doi:10.1371/journal.pone.0125273)
Supplement: S2 Table — (DOCX) [file pone.0125273.s002.docx]

**Supporting Information Table S2**. Atopy-related immunologic candidate genes/SNPs identified in the literature (see Supplementary Table 1) and subsequently selected (retained) for genotyping based on preliminary GWAS findings using pancreas cancer dbGaP datasets (n=59 genes, 152 SNPs)

| **Gene (n=59)** | **SNP (n=152)** |
| --- | --- |
| CCL18 | rs14304 |
|  | rs2735835 |
|  | rs1719220 |
| CCL3/CCL4 | rs1634508 |
| CLEC16A | rs3960630 |
| CSF2 | rs17674015 |
| DENND1B | rs12751508 |
|  | rs16841842 |
| DPP10 | rs6722852 |
|  | rs7421482 |
|  | rs980317 |
|  | rs998429 |
|  | rs6726425 |
|  | rs17728568 |
|  | rs272014 |
| EPX | rs12602891 |
| FCER1A | rs10489849 |
|  | rs2494264 |
| FCER1G | rs11421 |
|  | rs12094497 |
| FLG | rs10888470 |
|  | rs3126085 |
| GLI3 | rs3801182 |
|  | rs3801189 |
| HAVCR2 | rs13182119 |
| HLA-A | rs1611493 |
|  | rs2523809 |
|  | rs376646 |
| HLA-DQA2 | rs7774954 |
| HRH1 | rs6778270 |
| ICOS | rs4675377 |
| IKZF2 | rs10186029 |
|  | rs1871946 |
| IL10 | rs1800896 |
| IL13 | rs20541 |
| IL13RA1 | rs1316954 |
|  | rs2495622 |
|  | rs2495624 |
|  | rs2495626 |
|  | rs2997052 |
| IL13RA2 | rs5988202 |
|  | rs7891628 |
|  | rs638376 |
| IL17RB | rs1025690 |
| IL1B | rs1143634 |
| IL1RL1 | rs917998 |
| IL25 | rs8014568 |
| IL4R | rs1801275 |
|  | rs1805015 |
| IL7R | rs6897932 |
| KIRREL3 | rs4553380 |
|  | rs7113465 |
| KIT | rs1008658 |
| LRP1B | rs10469560 |
|  | rs10496860 |
|  | rs10496915 |
|  | rs10928120 |
|  | rs12476507 |
|  | rs13016322 |
|  | rs13029426 |
|  | rs1349223 |
|  | rs1449477 |
|  | rs2052910 |
|  | rs4507038 |
|  | rs6719026 |
|  | rs1882164 |
|  | rs6753877 |
|  | rs6758126 |
|  | rs10166782 |
|  | rs4507039 |
|  | rs13415720 |
|  | rs4363973 |
|  | rs2029142 |
| LTC4S | rs6895902 |
| MYB | rs9321496 |
| NETO1 | rs11876415 |
|  | rs1484218 |
|  | rs1893459 |
|  | rs6566663 |
|  | rs9958794 |
|  | rs10871704 |
|  | rs1484217 |
| NFATC1 | rs11664153 |
| NFATC2 | rs3787189 |
|  | rs6021231 |
|  | rs6067805 |
|  | rs4811174 |
| NOD1 | rs10257280 |
|  | rs2907749 |
|  | rs1558070 |
| NOS3 | rs3918227 |
| NPSR1 | rs1833090 |
| ORMDL3 | rs7216389 |
| PDE4D | rs17721878 |
|  | rs11739293 |
|  | rs1423473 |
|  | rs4394088 |
|  | rs6897671 |
|  | rs7732908 |
|  | rs2938787 |
|  | rs13159422 |
|  | rs6877743 |
|  | rs6869495 |
| PHF11 | rs9568227 |
|  | rs967163 |
| PIK3AP1 | rs1172480 |
| PRNP | rs6084833 |
| RORA | rs11631432 |
|  | rs974828 |
|  | rs12913421 |
|  | rs2433023 |
|  | rs12443239 |
| SEMA6A | rs10067680 |
|  | rs254227 |
|  | rs154576 |
| SH2B3 | rs3184504 |
| SPINK5 | rs3764930 |
| STAT1 | rs12693591 |
|  | rs6751855 |
|  | rs6758866 |
|  | rs3771300 |
| STAT4 | rs1031509 |
|  | rs6738544 |
|  | rs7572482 |
| TAP1 | rs2071538 |
| TBX21 | rs4141183 |
| TGFB2 | rs10482751 |
| TGFBR2 | rs2043136 |
|  | rs3773650 |
|  | rs3773656 |
|  | rs12495646 |
|  | rs3773634 |
|  | rs3773644 |
|  | rs3773652 |
|  | rs5020833 |
|  | rs877572 |
| TLR4 | rs2737191 |
|  | rs10759930 |
| TLR6 | rs4833095 |
| TMEM108 | rs9854387 |
|  | rs1464401 |
| TMEM232 | rs7721004 |
|  | rs7734243 |
|  | rs6891701 |
| TRA | rs1800388 |
|  | rs3811259 |
|  | rs7146411 |
|  | rs741713 |
|  | rs7147975 |
| TRB | rs11768792 |
|  | rs2040366 |
|  | rs4470937 |
